# Anisotropy of Thin Foils Obtained from Microwave-Irradiated Poly(Vinyl Alcohol) Aqueous Solutions

**DOI:** 10.3390/polym11061072

**Published:** 2019-06-21

**Authors:** Cristina-Delia Nechifor, Magdalena Aflori, Dana-Ortansa Dorohoi

**Affiliations:** 1 Department of Physics, Machine Manufacturing and Industrial Management Faculty, “Gheorghe Asachi” Technical University of Iasi, 67 Dimitrie Mangeron Bvd, RO-700050 Iasi, Romania; cristina.nechifor@tuiasi.ro; 2 Petru Poni Institute of Macromolecular Chemistry, 41A Grigore Ghica Voda Alley, 700487 Iasi, Romania; 3 Faculty of Physics, “Alexandru Ioan Cuza” University, 11 Carol I Bvd., RO-700506 Iasi, Romania; ddorohoi@uaic.ro

**Keywords:** PVA, microwave, birefringence, anisotropy, polymer membranes

## Abstract

In this paper, poly(vinyl alcohol) (PVA) foils of comparable thickness were obtained by using 10 wt % PVA aqueous solutions exposed to microwave (MW) radiations for different times. The main goal of this paper is to identify the effects of MW irradiation on the induced optical birefringence of PVA stretched foils, since it is known that the changes in the chemical and physical properties of polymers induced by radiations can influence the asymmetry of their molecular structures from which the birefringence of polymers derives. The efficiency of the MW oven was estimated, and the contribution of sensible and latent heat and heat loss to the absorbed energy was discussed. The effects of MW irradiation, in terms of absorbed energy, were evidenced by using FTIR spectra analysis, contact angle measurements, scanning electron microscopy (SEM) images, and induced optical birefringence. The dehydration (cross-linking) of PVA in aqueous solution and the dependence of the anisotropy on the absorbed MW energy, stretching ratio, and the type of hydrogen bonds formed are discussed in this study.

## 1. Introduction

Poly(vinyl alcohol) (PVA) is a hydrophilic polymer with many applications in the industrial field, including its use to obtain new retarders and polarizers [1,2]. The obtaining of polarizers is very simple and implies low costs. It requires PVA to be dehydrated, colored, dried, heated to the temperature at which it becomes softened, and then stretched. A non-stretched polymer foil is usually characterized by small intrinsic optical anisotropy due to the random orientation of the different macromolecular chains [3]. When the polymer chains are oriented in a certain direction, the optical anisotropies no longer compensate each other, and the material becomes birefringent [3,4]. The optical birefringence of the stretched polymer foils offers important information about the order degree of the polymer chains [5].

The anisotropic PVA foils increase their birefringence in the stretching process [3,4]. This type of birefringence is called orientation birefringence [5]. Previous studies [1,3,6,7] revealed that a supplementary induced birefringence was added to the initial one in the stretching process of PVA. The anisotropy increased with the stretching degree and decreased with foil thickness. A saturation in the alignment of the polymer chains, for high stretching degrees, was proven [3]. The optical properties of PVA foils were modified and/or improved by using electromagnetic radiation exposure. The induced birefringence increased with the exposure time when PVA foils were exposed to UV and gamma radiations. The irradiation increased the mobility of the polymeric chains, resulting in an easier alignment, but some undesired processes like photo-degradation and photo-oxidation appeared [7,8,9].

The main goal of this research is to obtain PVA thin foils with improved optical properties and consequently with high values of birefringence, without deteriorating their physical and/or chemical properties. The method proposed in this study consists in MW irradiation. Due to the poor dielectric properties of polymers [10], the absorbed energy from the electromagnetic field cannot directly break chemical bonds, and it cannot induce severe degradation [11]. The use of water as a solvent for PVA was chosen because it absorbs and transmits the energy of the MW to the polymer. The experimental results can be used in fundamental research on the optical properties of PVA foils, which is important for obtaining various optical devices [2,12].

## 2. Experimental Section

In this paper, 10 wt % PVA aqueous solutions were prepared and exposed to MW radiations for different times; then the influence of radiations on the physico-chemical properties and on the induced optical birefringence of PVA stretched foils with comparable thickness was analyzed.

### 2.1. Materials

PVA (hydrolyzed 99%, with average *M*_w_ = 22,000), from Merck KGaA, Darmstadt, Germany, was used. PVA solutions of 10 wt % were prepared by dissolving PVA in 70 °C distilled water and stirring for 6 h. The mixed solutions were outgassed for 8–12 min at 10–15 Torr. 

### 2.2. Preparation of the Samples

The MW exposure of the PVA solutions was carried out by means of a 360 W domestic microwave oven (Robert Bosch Hausgerate GmHMunchen, Germany), 2.54 GHz. Equal volumes (30 mL) of PVA solutions were placed in glass vessels and were exposed to MW radiations for 60, 90, 120, and 180 s. 

To increase the reproducibility of the experiments, the same experimental conditions were maintained for all samples. In this way, the MW oven was warmed-up by heating 50 mL of water for 3 min before each experiment. After irradiation, the samples were left to cool down at room temperature, then cast on a glass plate and dried for three days in air at room temperature (27 °C). The temperature and weight of the samples were measured before and after the MW treatment.

### 2.3. Characterization

The contact angles of the PVA foils were measured with a KSV CAM 101 (KSV Instruments Ltd., Helsinki, Finland) equipped with a video camera, liquid dispenser, and drop-shape analysis software (KSV CAM Optical Contact Angle and Pendant Drop Surface Tension Software, version 3.99, KSV Instruments Ltd, Helsinki, Finland) using the sessile drop technique, with three test liquids, namely water, ethanol, and glycerol. Ten different regions of the sample surface were selected, taking into consideration the contact angle values of three measurements with an error of ±1° for each kind of liquid. The thermodynamic equilibrium was established by waiting for a fixed time (10 s) before recording the contact angle. This way, the effect of PVA dissolution on the measurements was considered equal for all samples.

The structural characterization of the PVA thin foils was performed by ATR-FTIR analysis using a Bruker Vertex 70 type spectrometer (Bruker Optik GmbH, Ettlingen, Germany). The morphology was analyzed using scanning electron microscopy (SEM) images achieved with a FEI machine type: QUANTA 200 SEM (FEI Company, Brno, Czech Republic).

MW oven efficiency, $\eta_{\mathrm{MW}}$, was measured using the method proposed by Sólyom et. al [11]. In this method, the efficiency of the oven was expressed as the ratio between the absorbed, $E_{\mathrm{abs}}$, and emitted energy, $E_{\mathrm{emit}}$, as in Equation (1).

(1) $$\eta_{\mathrm{MW}}=\frac{E_{\mathrm{abs}}}{E_{\mathrm{emit}}}\times 100\left(\%\right)$$

The emitted energies were 0.72, 1.08, and 1.44 kJ/mL, respectively. The result of calculating the formula in (2) was 2.16 kJ/mL:

(2) $$E_{\mathrm{emit}}=\frac{P\cdot t}{V}$$

where P is the MW oven total power (W), t is the exposure time (s), and V is the volume (mL) of the PVA solution.

The absorbed energy was considered by an energy balance of three components [11]:

(3) $$E_{\mathrm{abs}}=Q_{\mathrm{sensible}}+Q_{\mathrm{latent}}+Q_{\mathrm{heat}\;\mathrm{loss}}$$

where: $Q_{\mathrm{sensible}}$ is the sensible heat (kJ/mL) representing the amount of heat required to change a unit mass of a substance by one unit of temperature; $Q_{\mathrm{latent}}$ represents the latent heat of vaporization (kJ/mL); and $Q_{\mathrm{heat}\;\mathrm{loss}}$ is the heat loss from a sample vessel, determined using the relation [11] (4):

(4) $$Q_{\mathrm{heat}\;\mathrm{loss}}=-U\cdot A\cdot \left[\left(\frac{T_{0}-T_{b}}{2}-T_{a}\right)\cdot t_{b}+\left(T_{b}-T_{a}\right)\cdot \left(t_{f}-t_{b}\right)\right]$$

In Equation (4), the term *A* is the heat transfer area, *U* is the global heat transfer coefficient, $T_{0}$ is the initial temperature, $T_{a}$ is the ambient temperature, $T_{b}$ is the boiling temperature of samples, $t_{f}$ is the total microwave treatment time, and $t_{b}$ is the time required to reach boiling temperature.

The value of the constant UA product was calculated from the temperature decrease of five water vessels with different amounts of water (30–75 mL), which were cooled to room temperature in the microwave oven, without irradiation. The calculated value was U·A=−0.2062±6.41%. 

The thickness of the foils was measured using a micrometer screw gauge. The average thickness was evaluated using the measurements at 10 different points on the same foil.

The polymer foils were cut in the form of standard tensile test specimen, heated at a temperature between 42 and 45 °C, then stretched. The birefringence was evaluated using a Babinet Compensator for different stretching degrees. 

The birefringence of the stretched polymeric foils is expressed as the difference between the main refractive indices, which are usually different:

(5) $$\Delta n(\lambda )=n_{e}(\lambda )-n_{o}(\lambda )$$

where $n_{e}(\lambda )$ is the refractive index of the film for linearly polarized radiation, with the electric field intensity parallel to the stretching direction, and $n_{o}(\lambda )$ is the refractive index of the film for linearly polarized radiation, with the electric field intensity perpendicular to the stretching direction [12].

The stretching degree is expressed as the ratio of the semi axes of an ellipse in which a circle initially drawn on the polymer foil degenerates in the stretching process. The stretching ratio was calculated with the formula (6):

(6) γ=a/b

where *a* is the length of the large semi-axis, and *b* is the length of the small semi-axis of the ellipse.

### 2.4. Experimental Device

The experimental design of the device used for the birefringence measurement of PVA foils was discussed in detail in other papers [1,3,6,7]. Briefly, it consists of two identical crossed polarizers, *P* and *A*, an anisotropic layer (AL), and a Babinet Compensator (BC). The PVA stretched foil is the anisotropic layer (AL), which changes the polarization state of light when introduced between the crossed polarizers. The BC compensates the pathway introduced by the foil. The anisotropy of the stretched foil is obtained by correlating the interference order of the radiations in the standardization operation with the pathway introduced by BC.

## 3. Results and Discussions 

### 3.1. Microwave Oven Efficiency 

The efficiency of the MW oven, the emitted energy, and the components ($Q_{\mathrm{sensible}}$, $Q_{\mathrm{latent}}$, and $Q_{\mathrm{heat}\;\mathrm{loss}}$) of the absorbed energy are listed in Table 1. The efficiency is between 49% and 75% depending on the MW irradiation time and the processes involved. 

**Figure 1:**
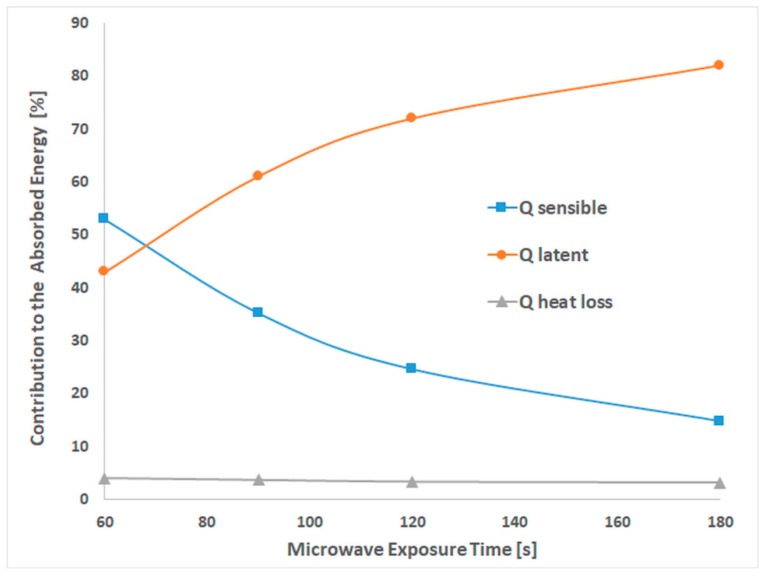
Contribution to the absorbed energy of the heat components versus MW exposure time.

The contributions of sensible and latent heat and heat loss to the absorbed energy are revealed in Figure 1. The heat-loss contribution is in the range of 3–4% of absorbed energy, with a very slow decrease with the exposure time.

The sensible and latent heat contribution to the absorbed energy is almost the same, 53% and 43%, when the exposure time was up to 68 s. The contribution of latent and sensible heat is dependent on MW time exposure. The latent heat increases concomitantly with the decrease of sensible heat, suggesting that water evaporation occurred [13] even after 68 s, when the boiling temperature was still not reached.

### 3.2. Spectral Analysis of PVA Foils

**Figure 2:**
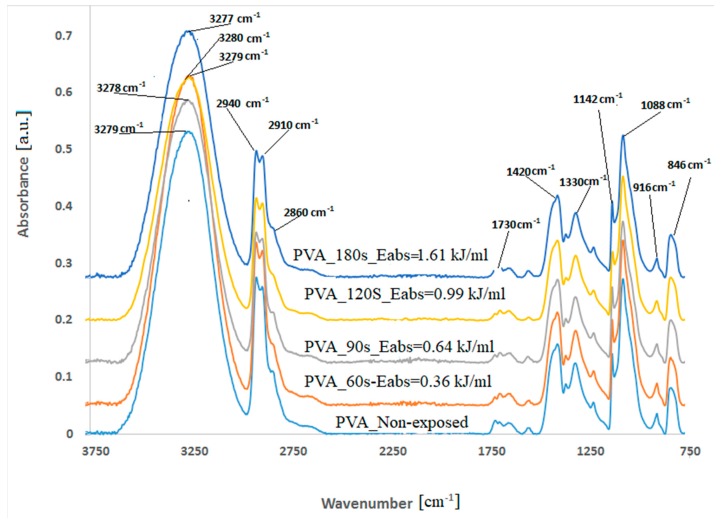
FTIR spectra of the obtained foils from the non-exposed and post-MW exposure PVA solutions.

The structural characterization of the samples was performed by FTIR analysis. Figure 2 presents the FTIR spectra of the foils obtained from the non-exposed and post-MW exposure solutions, revealing the major peaks associated with PVA.

The OH stretching vibration (ϑ = 3300 cm^−1^), the C–H broad alkyl valence band (ϑ = 2900–3000 cm^−1^), and the CH_2_ stretching (ϑ = 2850–2900 cm^−1^) [14,15,16] are mainly affected by the MW radiations. The intensities of OH stretching and of the C–H broad alkyl band decreased when the PVA aqueous solutions were irradiated for 90 and 120 s and were maintained almost the same with 180 s of MW exposure.

The presence of carbonyl groups, the peak from: ϑ = 1730 cm^−1^, in FTIR spectra of non-exposed PVA comes from the incomplete polymerization of vinyl acetate [16]. The intensity of the peak decreases for all the irradiated samples, suggesting a reduction in the number of residual acetate groups with exposure time. 

Sakura et al. [17] mentioned that the residual acetate groups decrease the tendency of PVA chains to self-associate by decreasing the regularity of the chain structure. By using PVA hydrolyzed to 99%, we reduce to a minimum the influence of the remaining 1% PVAc units on the intrinsic anisotropy of the foils. Exposure to MW radiation as a physical method for reducing the residual monomer content in the final polymer product [18] should lead to improvement in the symmetry of the polymer chains and, consequently, to an increase in the optical birefringence. 

In the bands from 1660 to 1570 cm^−1^, corresponding to the conjugated diones or single carbonyls in conjugation with C=C, no modifications were observed for the MW-exposed foils. This is possible if either no degradation appears or if the degradation is without isolated carbonyl formation [16], or if it is too small to be detected by FTIR analysis.

FTIR spectra of PVA foils revealed peaks from 1420, 916, and 846 cm^−1^, associated with the C–H bend of CH_2_, CH rocking, and C–O stretching, and the 1380–1330 cm^−1^ bands attributed to the combined frequencies of C–H and OH [15]. The stretching vibration of C–O from the C–O–C bond is ascribed to the peak from 1144 cm^−1^, and the stretching of C–O from the C–O–H bridge is present at 1088 cm^−1^ [16].

The increase in intensity of the 3300 cm^−1^ peak after 60 s of MW irradiation can be justified by the formation of hydrogen bonds with the water molecules [19]. 

The intensities of 2940, 2910, and 1088 cm^−1^ peaks increased. Considering the high flexibility of the PVA chain and the affinity of the polar groups to strongly absorb MW radiations [20], this aspect can be associated with segmental motion and group rotations in the relaxation processes [19].

Intra- and inter-molecular hydrogen bonds with water can be formed during heating. The intermolecular hydrogen bonds are formed by the syndiotactic sequences in PVA molecules, and the intramolecular hydrogen bonds result from the isotactic sequences [19]. Information about the syndiotactic structure of the analyzed PVA foils and the type of hydrogen bonds formed can be obtained from the frequency shift of the absorption peak at 3300 cm^−1^ and the intensity of the peak at 916 cm^−1^ [19,21].

In Figure 2, the wavenumber maximum IR absorption due to the OH stretching of the foils is noted next to the corresponding spectrum and is shifted when the PVA solutions are exposed to MW radiations. According to Nagura et al. [21], this phenomenon may arise due to structural changes occurring in the crystalline region of the PVA foils during irradiation. Exposure of PVA solutions to MWs leads to an initial decrease in frequency shift, when the samples are exposed up to 0.36 kJ/mL, and a high-frequency shift for a higher amount of the absorbed energies. The high-frequency shift indicates that the intramolecular hydrogen bonds are strengthened [21].

When the amount of absorbed energy is higher and water evaporation occurs, intramolecular hydrogen bonds form between the isotactic sequences of PVA chains. Absorption of an energy higher than 1kJ/mL leads to a low-frequency shift, lower than the initial value, indicating the development of intermolecular hydrogen bonds. These may cause a denser molecular packing, based on intermolecular rearrangements, and a restriction in the molecular motion [22].

Our observations and the results reported by Bernal et al. [16] and Petrova et al. [23] reveal degradation in the PVA chains during MW irradiation. The literature [16,23] reports a classical degradation mechanism of PVA by dehydration with cross-linking through ether bridges and the formation of double bonds and conjugated structures [23]. Since the appearance of carbonyl groups and conjugated systems was not evidenced in FTIR spectra, the possibility of the presence of ether bridges was studied. In this way, the ratios *R*_1_ and *R*_2_ were evaluated. 

*R*_1_ is the ratio between the peak area corresponding to the stretching vibrations of C–O–C (ϑ = 1144 cm^−1^) and the peak area corresponding to the stretching vibrations of C–O (ϑ = 846 cm^−1^). R_2_ is the ratio between the peak area corresponding to the stretching of C–O–H (ϑ = 1088 cm^−1^) and the peak area corresponding to the stretching vibrations of C–O. The peak area corresponding to the C–O stretching vibrations was chosen because no significant modification appeared during irradiation. The results obtained are listed in Table 2.

The *R*_1_ ratio decreased for 0.36 kJ/mL of absorbed energy, concomitantly with a strong increase in the R_2_ ratio. When the evaporation process occurred, the *R*_1_ ratio increased and *R*_2_ decreased with the exposure time. Starting with water loses from PVA solutions, degradation seems to occur. The first step evidenced in this study was the dehydration (cross-linking) of PVA with etheric (C–O–C) [20] bridge formation.

### 3.3. Contact Angle Measurements

Based on Fowkes theory [24,25], the values of the contact angles, measured with the three test liquids, were used to determine the surface polarity. In this method, the surface energy has two components: a dispersive and a polar one. The dispersive component is used to evaluate the work of adhesion, expressed as:

(7) $$W_{a}=2\sqrt{\gamma_{l}^{d}\gamma_{s}^{d}}+2\sqrt{\gamma_{l}^{p}\gamma_{s}^{p}}=\gamma_{l}\left(1+cos\theta \right)$$

where θ is the contact angle, the indices *l* and *s* are states of the test liquid and the polymer solid sample, while the indices *d* and *p* are associated with the disperse and polar components of the surface tension, γ.

To determine the surface tension of the PVA foils, the surface tension components of the test liquids were taken from the literature [26] and Equation (8) was applied.

(8) $$\gamma_{s}=\gamma_{s}^{d}+\gamma_{s}^{p},$$

The surface polarity was then evaluated using Equation (9):

(9) $$P=\frac{\gamma_{s}^{p}}{\gamma_{s}}=\frac{\gamma_{s}^{p}}{\gamma_{s}^{d}+\gamma_{s}^{p}}$$

To analyze the surface properties, the values of ethanol and glycerol contact angles, from Table 3, were introduced in Equation (7). The values obtained are displayed in Table 4.

All the contact angles of liquids increased after 180 s of irradiation, except for ethanol (Table 3). The water contact angle for PVA foils obtained from non-irradiated aqueous solutions is 6°. This decreases to 59° after 60 s of MW irradiation then increases to 70° after 180 s of exposure.

For all PVA foils, obtained before and after MW exposure of solutions, the dispersive component of surface tension is higher than its polar one. When the absorbed energy is 0.36 kJ/mL, the polar component decreases concomitantly with increases in the dispersive component, and the surface polarity decreases as well. For higher amounts of MW absorbed energy, the process is reversed. This means the polar component increases concomitantly with the decrease in the dispersive component and the surface polarity of the PVA foils also increases. This aspect is in good correlation with the spectral results, which showed the formation of intermolecular hydrogen bonds.

When a higher amount of MW energy is absorbed, and solvent evaporation occurs, the polar components increase, while the dispersive components decrease. A strong increase of surface polarity when the MW absorbed energy is above 0.36 kJ/mL sustains the phenomenon of dehydration and intermolecular rearrangements, which may have appeared as a first step in the degradation mechanism of PVA in aqueous solutions.

The surface free energy of hydration, $\Delta G_{w}$, was estimated to evidence the effects of MW radiations on the surface of PVA thin foils obtained from irradiated solutions. The critical value for $\Delta G_{w}$ is −113 mJ/M. It represents the equilibrium between hydrophilicity and hydrophobicity of the studied surface. For $\Delta G_{w}<-113\;mJ/m$, the examined surface can be considered hydrophilic, while, when $\Delta G_{w}>-113\;mJ/m$, it should be considered hydrophobic [27].

Equation (7) [25] was used for the determination of $\Delta G_{w}$. The contact angles of water, $\theta_{w}$, for the PVA foils were from Table 3, and the total surface tension of water, $\gamma_{l}$, was 72.8 mN/m [26].

(10) $$\Delta G_{w}=-\gamma_{l}\left(1+cos\theta_{w}\right)$$

**Figure 3:**
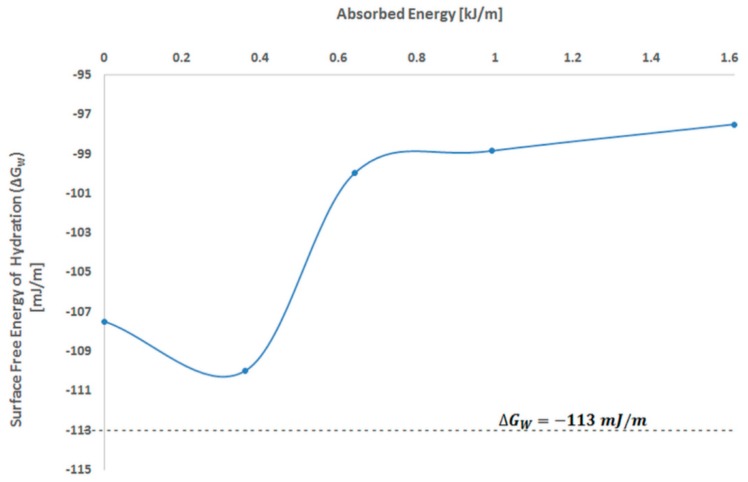
The dependence of surface free energy of hydration on MW absorbed energy.

The dependence of the surface free energy of hydration on the MW absorbed energy is presented in Figure 3.

From Figure 3, it results that for absorbed energy up to 0.36 kJ/mL, the value of $\Delta G_{w}$ decreases but does not reach the limit between hydrophilicity and hydrophobicity. The surface free energy of the hydration value increases with the amount of MW absorbed.

Thus, the results for the surface polarity (Table 4) of PVA foils and the surface free energy reveal that the hydrophilicity of the studied samples decreases, which is enhanced as the MW irradiation increases. The surface polarity increases for a high quantity of MW absorbed radiation. This may be attributed to the orientation and disorientation of permanent dipoles of the molecules from the polymer chains [20]. When the temperature is enough for water evaporation, the cross-linking of PVA chains through etheric and esteric bridges and intramolecular hydrogen bonds occurs, causing a different molecular packing. In this way, the surface of the foils obtained from MW-irradiated PVA solutions contains more polar groups than the foils obtained from non-exposed solution.

### 3.4. Scanning Electron Microscopy

**Figure 4:**
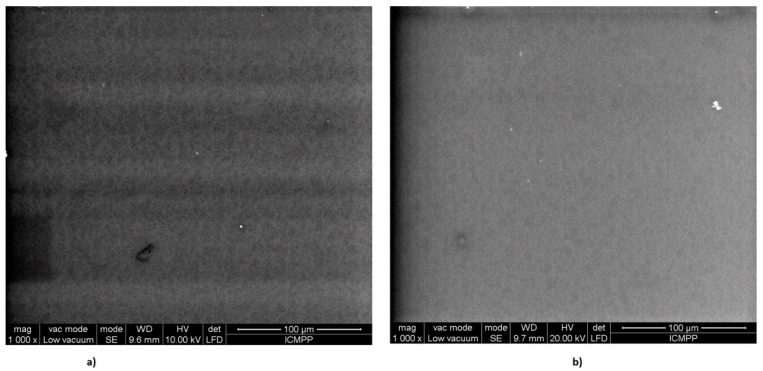
SEM images of PVA foils obtained (**a**) before and (**b**) after 180 s of MW irradiation of aqueous solutions.

The morphology of the PVA thin foils was investigated using scanning electron microscopy (SEM). SEM images of PVA foils obtained before and after 180 s of MW irradiation of aqueous solutions are given in Figure 4.

From Figure 4a,b, we see that no significant modification in surface morphology appeared when the PVA solution was exposed to MWs. However, a denser packaging of PVA fibers can be noticed for the sample irradiated for 180 s. 

### 3.5. Anisotropy of PVA Thin Foils

**Figure 5:**
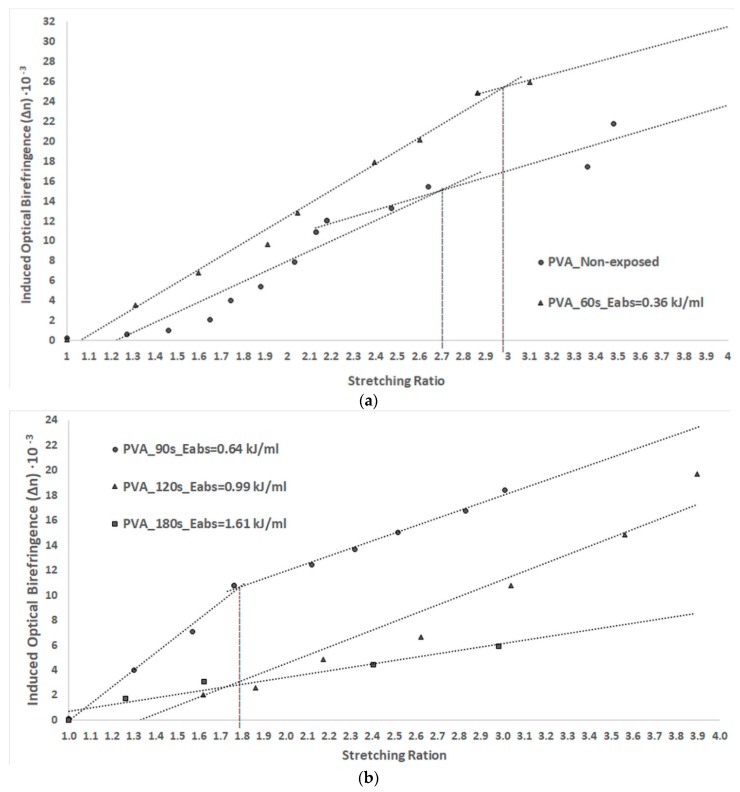
(**a**) Dependence of induced optical birefringence versus stretching ratio of PVA foils obtained before and after 60 s of MW irradiation; (**b**) Dependence of induced optical birefringence on the stretching ratio of PVA foils obtained after 90, 120, and 180 s of MW irradiation.

The dependence of the induced optical birefringence on the stretching ratio is represented in Figure 5a for PVA foils obtained before and after 60 s of MW irradiation and in Figure 5b for the foils obtained after 90, 120, and 180 s of MW exposure.

The graphs in Figure 5a,b show that the induced optical birefringence increases with the stretching ratio for all samples, and it is dependent on the MW absorbed energy. Improved optical birefringence can be observed for samples MW irradiated for 60 and 90 s. The absorption of a small amount of energy results in heating the samples without evaporation. It can be observed that intermolecular bonds are formed by the syndiotactic sequences of PVA; moreover, the chains have a tendency of associating in such a way that surface polarity decreases. The anisotropy of the foils increased because of a higher order degree of the irradiated polymer compared to that of the unexposed sample. 

The birefringence’s tendency to increase linearly with the stretching ratio is also evidenced. The slopes of the linear dependence decrease when the stretching ratio is above a specific value, depending on the absorbed MW energy. 

The slope value gives information about the mechanism of polymeric chain alignment during the stretching process [3,7]. When the slope value starts to decrease after a stretching ratio, it indicates that the capacity of polymeric chains to align after the stretching direction and their mobility decreases. The stretching ratio at which the slope of the line changes was evaluated and is indicated in Figure 5a,b.

The stretching ratio at which the slope of the line changes is 2.7 for the unirradiated sample, while 2.95 and 1.75 are the values obtained for the PVA foil attained from solutions exposed for 60 and 90 s, respectively. 

The slope of linear dependence between the birefringence and stretching ratio of the foils obtained for the solutions exposed to MWs for 120 and 180 s was smaller than for the foils exposed to a lower amount of MW energy, and no change in its value was observed. These aspects suggest that the saturation of the chain alignment for these foils was not achieved for stretching ratios smaller than 4. The polymeric chains of these films possess a diminution of their flexibility. This phenomenon is possible due to the dense packaging of PVA chains because of dehydration and intermolecular rearrangements appearing during the irradiation process.

The anisotropy of the sample exposed for 60 and 90 min is smaller than for the PVA foils obtained from unirradiated solution. This aspect confirms that a quantity of MW absorbed energy lower than 0.69 kJ/mL may lead to improvements in the chemical and physical properties of PVA foils.

Foils with enhanced anisotropy were obtained when the MW absorbed energy led to the appearance of intermolecular hydrogen bonds. The association of PVA chains occurs in such a way that the polarity and surface free energy of hydration decrease and are lower than those of the unirradiated sample.

The cross-linking of PVA chains through etheric/esteric bridges and the appearance of intramolecular hydrogen bonds causing a denser molecular packing with a limitation in the chain motion [21] decreased the induced optical birefringence of the PVA foils. The chain association when evaporation occurs as polar groups are found on the surface of the films. The anisotropy of these films is lower than that of the unirradiated ones.

## 4. Conclusions

In this study, PVA aqueous solutions were prepared and MW irradiated to modify the optical properties of the thin foils. MW irradiation seems to be a promising method in PVA processing since it does not induce severe polymer degradation.

The results obtained suggest that the processes involved in MW irradiated PVA aqueous solutions are dependent on a critical point. This point is represented by the time of exposure or by the absorbed energy at which solvent evaporation occurs. For an exposure time below 68 s, the effects of MWs on the PVA solution consist in enhancing the molecular mobility and in the appearance of intermolecular hydrogen bonds. The residual acetate groups are reduced during MW irradiation and the order degree of the polymer chains increase. 

When the phenomenon of solvent evaporation occurred, the cross-linking of PVA chains through esteric bonds, etheric bridges, and intramolecular hydrogen bonds were noticed, causing a compact molecular packing, with more polar groups oriented at the surface of the PVA foils.

PVA thin foils with improved anisotropy, and consequently with high values of birefringence, without deteriorated physical and chemical properties were obtained when the PVA aqueous solutions were MW irradiated for 60 s with an amount of absorbed energy below 0.69 kJ/mL.

The intermolecular hydrogen bonds may lead to improvement in the optical birefringence of PVA foils, while the presence of intramolecular hydrogen bonds reduces the optical properties of polymeric films. 

## Figures and Tables

**Table 1:** Energy distribution in the microwave (MW) irradiation of poly(vinyl alcohol) (PVA) solutions as a function of the exposure time.

	MW Time (s)	$E_{emit}$ (kJ/mL)	$Q_{sensible}$ (kJ)	$Q_{latent}$ (kJ/mL)	$Q_{heat\;loss}$ (kJ)	$E_{abs}$ (kJ/mL)	$\eta_{MW}$ (%)
1	60	0.72	5.59	4.54	0.43	0.36	49
2	90	1.08	6.79	11.80	0.72	0.64	59
3	120	1.44	7.28	21.34	1.01	0.99	69
4	180	2.16	7.14	39.73	1.57	1.61	75

**Table 2:** Ratios R_1_ and R_2_ as a function of the absorbed MW energy.

Exposure Time (s)	$E_{abs}$ (kJ/mL)	$R_{1}\cdot 10^{-2}$	$R_{2}$
0	0	22.39	2.27
60	0.36	22.27	2.31
90	0.64	22.31	2.19
120	0.99	22.44	2.18
180	1.61	23.14	2.24

**Table 3:** The contact angles of water, glycerol, and ethanol for PVA foils obtained from aqueous solutions exposed to MWs for different times.

Exposure Time (s)	$E_{abs}$ (kJ/mL)	Contact Angle (°)		
		Water	Ethanol	Glycerol
0	0	62	21	56
60	0.36	59	13	53
90	0.64	68	12	57
120	0.99	69	13	63
180	1.61	70	19	66

**Table 4:** The surface tension components of PVA foils obtained before and after exposure of solutions to MW radiation, for different absorbed energies.

Exposure Time (s)	$E_{abs}$ (kJ/mL)	$\gamma_{s}^{p}$ (mN/m)	$\gamma_{s}^{d}$ (mN/m)	$\gamma_{s}$ (mN/m)	P (%)
0	0	0.86	86.58	87.44	0.99
60	0.36	0.80	92.60	93.40	0.86
90	0.64	1.97	71.57	73.54	2.68
120	0.99	5.20	41.47	46.67	11.14
180	1.61	5.69	36.54	42.23	13.47
